# Awareness, attitudes and acceptability of the HPV vaccine among female university students in Morocco

**DOI:** 10.1371/journal.pone.0266081

**Published:** 2022-04-08

**Authors:** A. Yacouti, N. Elkhoudri, A. El got, A. Benider, F. Hadrya, R. Baddou, A. Forster, M. Mouallif

**Affiliations:** 1 Laboratory of Health Sciences and Technologies, Epidemiology and Biomedical Unit, Higher Institute of Health Sciences, Hassan First University of Settat, Settat, Morocco; 2 Faculty of Medicine and Pharmacy, Hassan II University, Casablanca, Morocco; 3 Department of Behavioural Science and Health, Institute of Epidemiology & Health, University College London, London, United Kingdom; Middle East Liver Diseases (MELD) Center, Tehran, Iran, ISLAMIC REPUBLIC OF IRAN

## Abstract

**Background:**

In Morocco, cervical cancer is the second most common cancer affecting women behind breast cancer. The Human PapillomaVirus (HPV) vaccine has been available in Morocco since 2008 but its introduction in the national immunization program is still under discussion. There is limited data regarding acceptability and predictors of HPV vaccine acceptability among Moroccan young women. This study aimed to evaluate the awareness of female university students of HPV and the vaccine and to identify predictors of HPV vaccine acceptability.

**Methods:**

We conducted a structured interviewer-administered questionnaire with 1087 participants in six Moroccan universities between May 2019 and June 2020.

**Results:**

The awareness of HPV infection was 14.7% and of HPV vaccine was 7.8%. The rate of immunization coverage against HPV was less than 1%. Over 67% of participants were willing to receive the HPV vaccine. Awareness of cervical cancer (*p-Value* = 0.04) and the HPV vaccine (*p-Value* = 0.01), and acceptability of Pap smear test (*p-Value* <0.01) were significant predictors of HPV vaccine acceptability.

**Conclusions:**

This study revealed an insufficient amounts of awareness of HPV and of HPV vaccine uptake in a sample of Moroccan university young women. This lack of awareness needs intervention, and it is important to develop an awareness program for young female population either within or outside universities in order to promote vaccination uptake and ultimately lower the cervical cancer rate in Morocco.

## 1. Introduction

Cervical cancer is the second most common cancer behind breast cancer affecting women worldwide, with approximately 570 000 new cases and 311 000 deaths annually [1]. In Morocco, cervical cancer is the second women’s cancer behind breast cancer, with an estimated 3388 new cases and 2465 deaths annually [1].

Human PapillomaVirus (HPV) infection is a common sexually transmitted infection. Virtually all cases of cervical cancer are linked to infection with high-risk oncogenic HPV [2]. The last decade has been marked by the introduction of a vaccine against this infection and over 80 countries have introduced the HPV vaccine into their national immunization program [3]. Although cervical cancer is one of the few preventable human cancers, its incidence is significantly high in developing countries [4].

Due to the high prevalence of HPV infection in girls from their first sexual intercourse [5], the most national program of different countries targeted young adolescent girls [6]. However, the specific age groups targeted vary from one country to another [6].

The possibility of preventing cervical cancer through vaccination is both an invaluable opportunity and a difficult challenge. A critical first step to tailoring strategies that can be adopted to increase HPV vaccine uptake is to understand the predictors of the acceptability of the HPV vaccine.

Many investigations have revealed a variable level of awareness, attitudes and acceptability of young populations towards the HPV vaccine in different countries [7, 8]. In Morocco, limited data is available regarding this subject. The only study done in a population of young Moroccans of both genders revealed a low level of acceptability of the HPV vaccine. However, the survey included males, which limits our understanding of the acceptability of the HPV vaccine among women (only 256 females took part of the survey). Moreover, the female population in this study included adolescents, a population of which vaccination decisions still depending on parents [9]. Thus, in the present study we aimed to better investigate this subject by carrying out a survey exclusively in female university students, a population more likely to be making immunization decisions themselves [10]. We specifically aimed to evaluate awareness of participants regarding cervical cancer and its prevention, and to identify predictors of HPV vaccine acceptability.

## 2. Methods

### 2.1. Study design

A cross-sectional study was conducted using a structured interviewer-administered questionnaire among female students between May 2019 and June 2020, in six universities among the twelve Moroccan public universities. This was within five various regions among the twelve Moroccan regions. A Purposive sampling technique was employed to choose regions to ensure that the sample is equally balanced across different Moroccan regions. The five regions are as follow: Souss-Massa (Agadir: southern atlantic of Morocco), Casablanca-Settat (Casablanca: center of the country and the economical capital, Settat: dominance of the rural population), Rabat-Salé-Kénitra (Rabat: the administrative capital), Marrakech-Safi (Marrakech: fourth largest city and the touristic capital located in the south of Morocco), and Tanger-Tétouan-Al Hoceïma (Tanger: the north west of Morocco). Quota sampling was used to calculate the representative sample size of each selected university. The choice of individuals was arbitrarily made within institutions belonging to each university. The interviews were conducted individually, in a separate lecture room within each university institution. Participants were invited to take part in this study after getting their consents and if they were Moroccans and aged between 17 and 26 years old.

### 2.2. Data measurement

A multidisciplinary staff developed the questionnaire, in English, based on previous studies [11–13]. The questionnaire was translated into Moroccan dialect Arabic, and was then back translated into the original language. Lastly, the questionnaire was piloted on a sample of thirty participants. The questionnaire is composed of three different parts; i) socio-demographic characteristics, ii) awareness of cervical cancer and HPV infection, and iii) awareness, attitude, and acceptability of HPV vaccine (S1 Fig in S1 File). To assess the level of awareness of cervical cancer, participants were asked if they had already heard of cervical cancer. Those who were aware of cervical cancer, were then asked if they had ever heard of HPV. After providing all participants with accurate information on cervical cancer HPV and Pap smear test, we asked them if they had ever heard previously of Pap smear test and HPV vaccine to evaluate their level of awareness of these two cervical cancer preventive measures. To evaluate the acceptability of HPV vaccine within the whole study population, we first provided all participants with accurate information about the HPV vaccine. We then evaluated whether they were willing to get vaccinated in the future. Participants who were reluctant to get the HPV vaccine in the future were asked to freely express why this was the case.

### 2.3. Statistical analysis

Data were analyzed via SPSS v 25. We carried out a descriptive analysis to describe the socio-demographic characteristics and the level of awareness of participants. To identify the attitudinal, awareness and socio-demographic predictors of HPV vaccine acceptability, we used the univariate logistic regression. We then used a multivariate model to assess the net effect of each socio-demographic and attitudinal covariate on acceptability of the HPV vaccine. Association was considered statistically significant at *p*<0.05. The 95% confidence interval was used to estimate the precision of the Odds Ratio (OR). The multivariate model was evaluated using the Hosmer-Lemeshow goodness-of-fit test.

### 2.4. Statement of ethics

Participants were informed that the survey was anonymous and they all had volunteered to participate in the study. The study protocol was approved by the national committee on research ethics of the Faculty of Medicine and Pharmacy of Rabat, University Mohammed V (ref. 58/19). The national committee of the protection of personal data (*CNDP)* authorized the implementation of the survey.

## 3. Results

### 3.1. Socio-demographic characteristics of participants

We approached 1145 university women, among them 1087 completed the interview. The response rate was 95%. A summary of the socio-demographic characteristics of participants is provided in Table 1. The average age was 20.22 (range 17–26). The majority of participants followed non-health related studies (71.9%) and they were undergraduate students (88.3%). More than half of participants (58.4%) declared a monthly family income greater than 5000 MAD (Moroccan Dirham) which represent a medium income for Moroccan population (5000 MAD = US $500). About half of respondents (49%) reported that their fathers had a high educational level and almost 42% of participants declared that their mothers were unschooled.

**Table 1:** Socio-demographic characteristics of participants by HPV vaccine acceptability. Morocco between May 2019 and June 2020 (N = 1087*)*.

Categorical variables	Total sample of participants (N = 1087)	Univariate analysis		Multivariate analysis	
		OR (95% CI)	p-Value	OR (95%CI)	p-Value
**Age**					
17–21	76.4 (830)	1		1	
22–26	23.6 (257)	0.77 (0.58–1.04)	0.09	0.83 (0.60–1.13)	0.25
**Field of study**					
Non Health-related	71.9 (782)	1		1	
Health-related	28.1(305)	1.75 (1.29–2.36)	<0.01	1.22 (0.87–1.71)	0.24
**Year of study**					
Bachelor	88.3 (960)	1			
Masters-Doctorate	11.6 (127)	0.88 (0.60–1.31)	0.54		
**Living area**					
Rural area	10.6 (115)	1		1	
Urban area	89.4 (972)	0.68 (0.43–1.05)	0.08	0.66 (0.42–1.05)	0.08
**Income**					
< = 2566 MAD	8.9 (97)	1		1	
2567–5000 MAD	19.4 (211)	0.79 (0.74–1.33)	0.38	0.83 (0.48–1.41)	0.49
>5000 MAD	58.4 (635)	1.05 (0.66–1.67)	0.81	0.97 (0.60–1.56)	0.90
I don’t want to answer‎/I don’t know	13.2 (144)	0.70 (0.40–1.21)	0.20	0.62 (0.35–1.10)	0.10
**Father’s educational level**					
No schooling	20.1 (219)	1		1	
Elementary school	17.9 (195)	1.09 (0.73–1.62)	0.66	1.19 (0.78–1.80)	0.41
Secondary school	12.6 (137)	1.42 (0.91–2.23)	0.11	1.66 (1.02–2.70)	0.03
High school and more	49.3 (536)	1.97 (1.41–2.74)	<0.01	1.73 (1.13–2.67)	0.01
**Mother’s educational level**					
No schooling	41.9 (455)	1		1	
Elementary school	12.5 (136)	0.99 (0.67–1.48)	0.99	0.84 (0.55–1.29)	0.44
Secondary school	11.0 (120)	0.96 (0.63–1.46)	0.87	0.79 (0.49–1.26)	0.32
High school and more	34.6 (376)	1.87 (1.73–2.53)	<0.01	1.18 (0.78–1.78)	0.42

### 3.2. Cervical cancer, Pap smear test and HPV awareness

Only 18.1% of participants were unaware of cervical cancer. Over 74% of participants who were aware of cervical cancer reported that they knew that it can be screened at an early stage. The majority of those who were aware of cervical cancer were unaware of HPV (85.3%). Most participants, 82.8% and 92.2% were, respectively, unaware of the Pap smear test and the HPV vaccine (Table 2).

**Table 2:** Participants’ awareness of cervical cancer, HPV, Pap smear testing and HPV vaccine by willingness to get the HPV vaccine in the future. Morocco between May 2019 and June 2020.

Categorical variables	Total sample of participants (N = 1087)	Univariate analysis		Multivariate analysis			
		OR (95% CI)	p-Value	OR (95% CI)		p-Value	
**Heard of cervical cancer? (N = 1087)**							
No	18.1 (197)	1		1			
Yes	81.9 (890)	1.56 (1.13–2.14)	<0.01	1.40 (1.00–1.96)		0.04	
**The risk of getting cervical cancer (n = 890)** ^**1**^							
Below average	35.5 (316)	1					
Average	32.7 (291)	1.03 (0.72–1.46)	0.86				
Above average	13.4 (119)	0.97 (0.61–1.54)	0.90				
I don’t know	18.4 (164)	0.64 (0.43–0.96)	0.03				
**Cervical cancer can be detected at an early stage?(N = 890)** ^**1**^							
No	10.1 (90)	1					
Yes	74.7 (665)	1.28 (0.80–2.04)	0.29				
I don’t know	15.2 (135)	0.98 (0.56–1.72)	0.95				
**Cervical cancer can often be cured?(n = 890)** ^**1**^							
Strongly agree	(26.6) 237	1					
Agree	(46.3) 412	0.89 (0.63–1.28)	0.55				
Disagree	(8.2) 73	1.07 (0.59–1.94)	0.81				
Strongly Disagree	(8) 71	0.54 (0.31–0.95)	0.03				
I don’t know	(10.9) 97	0.64 (0.38–1.05)	0.08				
**A diagnosis of cervical cancer is a death sentence?(n = 890)** ^**1**^							
Strongly disagree	(73.8) 657	1					
Disagree	(14.9) 133	0.71 (0.48–1.06)	0.09				
Agree	(3.5) 31	0.41 (0.20–0.86)	0.01				
Strongly agree	(1.7) 15	1.56 (0.43–5.61)	0.49				
I don’t know	(6.1) 54	0.61 (0.34–1.09)	0.09				
**Heard of HPV (n = 890)** ^**1**^							
No	85.3 (759)	1					
Yes	14.7 (131)	1.13 (0.75–1.70)	0.55				
**HPV can be transmitted sexually? (n = 131)** ^**2**^							
No‎/ I don’t know	35.1 (46)	1					
Yes	64.9 (85)	1 (0.45–2.22)	0.99				
**Heard of Pap smear test (N = 1087)**							
No	82.8 (900)	1					
Yes	17.2 (187)	0.92 (0.66–1.29)	0.65				
**Willing to get a Pap smear in the future (N = 1087)**							
No	17.8 (193)	1			1		
Yes	82.2 (894)	2.54 (1.85–3.49)	<0.01		2.47 (1.77–3.45)		<0.01
**Heard of HPV vaccine (N = 1087)**							
No	92.2 (1002)	1			1		
Yes	7.8 (85)	3.11 (1.66–5.80)	<0.01		2.38 (1.22–4.65)		0.01

^1^: Number of participants who are aware of cervical cancer.

^2^: Number of participants who are aware of cervical cancer and aware of HPV.

### 3.3. HPV vaccine and Pap smear test acceptability

Over 67% of participants were willing to receive the vaccine. The high cost of the vaccine was cited as the main barrier to willingness to get the HPV vaccine for those who were unwilling to receive the vaccine (48.1%) (Table 3). Furthermore, almost 83% of participants intended to have a regular Pap smear test in the future (Table 2).

**Table 3:** The major barriers to willingness to get the HPV vaccine. Morocco between May 2019 and June 2020.

	N	%
Major barriers	351*	
High cost	169	48.1
Concern about side effects	52	14.8
I need my parent’s approval	28	8
I need medical prescription	23	6.6
I am against vaccination in general	11	3.1
I am not convinced of efficacy of HPV vaccine	32	9.1
Not interesting to me	36	10.3

*Number of participants who have not expressed their willingness to receive the HPV vaccine.

### 3.4. Predictors of willingness to get the HPV vaccine in the future

#### 3.4.1. Socio-demographic predictors

The univariate logistic regression showed that participants’ current field of study at university and parent’s educational level were associated with whether they were willing to get the HPV vaccine in the future. The participants who followed health-related studies were more likely to say they were willing to get the vaccine in the future compared to those who followed non-health related studies (OR:1.75, 1.29–2.36 CI 95%). Participants whose mothers had a high level of education were more likely to be willing to get the HPV vaccine in the future compared to those with unschooled mothers (OR:1.87, 1.73–2.53 CI 95%). Participants whose fathers had a high level of education were more likely to be willing to get the HPV vaccine in the future compared to those with unschooled fathers (OR:1.97, 1.41–2.74 CI 95%) (Table 1). Results of multivariate logistic regression showed that no demographic variables were significantly associated with willingness to get the HPV vaccine in the future (Table 1).

#### 3.4.2. Attitudinal and awareness predictors

The univariate logistic regression revealed that awareness of cervical cancer and of the HPV vaccine, willingness to get the Pap smear test in the future, and believing that cervical cancer is a deadly cancer were associated with willingness to get the HPV vaccine in the future. Participants who were aware of cervical cancer were more likely to be willing to get the HPV vaccine in the future compared to those who were unaware of this cancer (OR:1.56, 1.13–2.14 CI 95%). Participants who were willing to have a Pap smear test in the future were more willing to get the HPV vaccine in the future than those who were unwilling to have a Pap smear test in the future (OR: 2.54, 1.85–3.49 IC 95%). Participants who were aware of HPV vaccine were more willing to get the vaccine in the future than those who had never heard of (OR: 3.11, 1.66–5.80 CI 95%) (Table 2). Data related to multivariate logistic regression showed that awareness of cervical cancer, HPV vaccine, and willingness to have a Pap smear test in the future were associated with willingness to get the HPV vaccine in the future (Table 2).

## 4. Discussion

This research aimed to investigate awareness and attitudes towards cervical cancer prevention, as well as the correlates of willingness to get the HPV vaccine in the future among female university students in Morocco.

A high proportion of participants were aware of cervical cancer (82%), which is similar to a previous study conducted in a comparable country; 69% of Lebanese students were aware of cervical cancer [14]. However, awareness was slightly lower than what was revealed in the study conducted by Mouallif *et al* [11] where 93.7% of mothers had heard of cervical cancer. This could be explained by the fact that the current ministry of health program aiming the early detection of cervical cancer does not reach young women. Indeed, a pilot program of education and awareness towards cervical cancer targeting the young population is highly needed.

Despite this high level of awareness of cervical cancer, few people were aware of HPV, the cause of cervical cancer. Indeed, only 15% of participants who were aware of cervical cancer were aware of HPV. A similar figure was reported in a study of ethnically diverse female university students (21.7%) [15]. However, a far higher level of awareness of HPV (59.89%) was reported in a previous study conducted within a young population in Mainland China which is also considered as a developing country [16]. A higher levels of awareness of cervical cancer have been reported in college students in South Carolina (95.3%) [13]. This discrepancy could be closely associated to the availability of awareness campaigns in South Carolina, supporting that there is a need of awareness campaigns for Moroccan young females, which is in line with the National Cancer Prevention and Control Plan recommendations [17].

Awareness of the Pap smear test was also low, with only 17% of participants having heard of the test. Awareness among these Moroccan students was lower than what was revealed within a population of Muslim women in other Arab countries [18]. However, a previous Moroccan study conducted among a population of parents have revealed that 44.6% of mothers were aware of Pap smear test [11]. This result could be explained by the fact that mothers have benefited from a sensitization towards this test during their gynecological consultations. Nevertheless, the majority of participants (82%) expressed their willingness to undergo the Pap smear test in the future. This suggests, once again, that there is an urgent need to implement an educational program for cervical cancer suitable for young women.

Overall, the results showed very poor level of awareness of the HPV vaccine (8% had heard of it), which is likely related to the absence of education on HPV vaccine, and a national vaccine program. Moroccan parents have equally been found to have very low awareness of the HPV vaccine (14.3%) [11]. However, a study done in college students in South Carolina showed that more than 90% of them were aware of HPV vaccine, and this level of awareness was explained by the impact of multicomponent public health campaigns to promote cervical cancer prevention [13]. Furthermore, a recently published study on awareness of HPV vaccine within college students in China based on meta-analysis, concluded that awareness of HPV vaccine of a population of college students was relatively high in European countries compared with China [19]. These results underline the need to raise awareness of HPV and cervical cancer prevention in developing countries.

All but one of those who were aware of the HPV vaccine within our study population were unvaccinated. This finding was not surprising considering the absence of HPV vaccine in the national immunization program and the lack of public awareness campaigns to promote its acceptability. In this regard, a study conducted among a population of medical students in Lebanon reported also a low level of HPV vaccine uptake [20].

Interestingly, in the present study more than 67% of participants were willing to receive the HPV vaccine. A similar investigation conducted in Saudi Arabia reported similar figure; 64.3% of Saudi women were willing to receive the HPV vaccine [7]. However, a prior Moroccan survey conducted in 2015 with adolescents and young adults of both genders reported a low level of HPV vaccine acceptability (27%) [9]. The low level of acceptability in the later Moroccan study, could be mainly related to the fact it was evaluated either in males who may be less willing to have the vaccine than females.

In respect to the major barriers to willingness to get the HPV vaccine, our study showed that the cost of vaccine led to a negative intention to receive the HPV vaccine. This result corroborates with the finding of a meta-analysis done across South East Asian and Western Pacific regions [21]. However, others barriers have been raised more prominently in other research compared to this research including lack of perceived risk for cervical cancer, logistical barriers and the concerns regarding safety and efficacy of the vaccine [8, 15].

Multivariate logistic regression revealed significant positive associations between willingness to get the HPV vaccine and awareness of this vaccine, and of cervical cancer. This finding was supported by many previous studies [22]. Moreover, our study revealed a positive association with willingness to get a Pap smear test in the future and willingness to get the HPV vaccine. Mothers’ screening behavior has previously been shown to be associated with their daughters’ vaccination acceptability [23] and in another study, girls of mothers who were engaged in the cervical screening program were more likely to initiate and complete the vaccination [24]. All these considerations point to the importance of education and awareness campaigns on cervical cancer prevention, with the aim of promoting willingness to attend for screening and get the vaccine. It is also recommended to implement an information system for the whole population and promote advocacy for cancer prevention mobilization [17].

Our study did not find any relationship between socio-economic background and HPV vaccine acceptability. Monthly income has been found to be positively associated with HPV vaccine acceptability in a previous Moroccan study, although done within a population of parents [11], and a systematic review focusing on European countries has also shown that low socio-economic status was correlated with individuals not getting the vaccine [25].

### 4.1. Strengths and limitations

Our study included a relatively large sample size and the study population was exclusively female (the group who are eligible for cervical cancer prevention program). Our investigation constitutes the first Moroccan study evaluating awareness of, attitudes towards, and factors associated with willingness to get the HPV exclusively in a population of young adult women. We used a structured interviewer-administered questionnaire for data collection whose results are described to be more reliable than self-administered interviewing [26]. We used non-probabilistic sampling which limits the generalization of our findings and our sample was limited to mainly urban dwelling students.

## 5. Conclusions

The awareness regarding HPV and HPV vaccine of female Moroccan university students seems to be not sufficient. The rate of vaccination coverage against HPV was almost zero. However, the vast majority of our study population was receptive to future HPV vaccination. Hence, the lack of awareness needs intervention, Therefore, developing an educational program on cervical cancer prevention seems to be a priority. The current rate of vaccine uptake is not sufficient in the absence of a vaccination program.

## Supporting information

S1 File Questionnaire used for data collection. (DOCX)
