# Supplementary material for: Awareness, attitudes and acceptability of the HPV vaccine among female university students in Morocco
Source: PLoS One. 2022 Apr 8;17(4):e0266081. doi: 10.1371/journal.pone.0266081 (PMC8993020; doi:10.1371/journal.pone.0266081)
Supplement: S1 File — (DOCX) [file pone.0266081.s001.docx]

***Awareness, attitudes and acceptability of the HPV vaccine among female university students in Morocco***

A Yacouti^1^, N Elkhoudri^1^, A El got^1^, A Benider^2^, F Hadrya^1^, R Baddou^1^, A Forster^3¶^, M Mouallif ^1^*^¶^

^1^Laboratory of Health Sciences and Technologies, Epidemiology and biomedical unit, Higher Institute of Health Sciences, Hassan First University of Settat, Settat, Morocco.

^2^Faculty of Medicine and Pharmacy, Hassan II University, Casablanca, Morocco

^3^Department of Behavioural Science and Health, Institute of Epidemiology & Health, University College London, London, United Kingdom.

* Corresponding author

E-mail : m.mouallif@gmail.com (MM)

¶These authors contributed equally to this work.


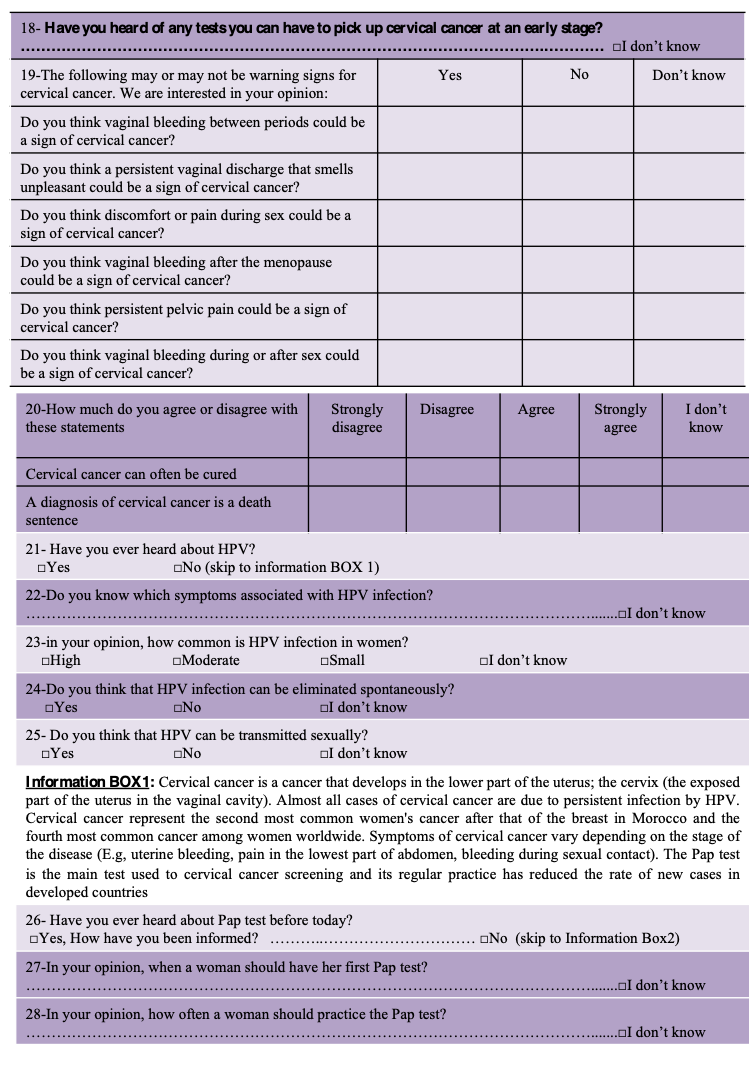

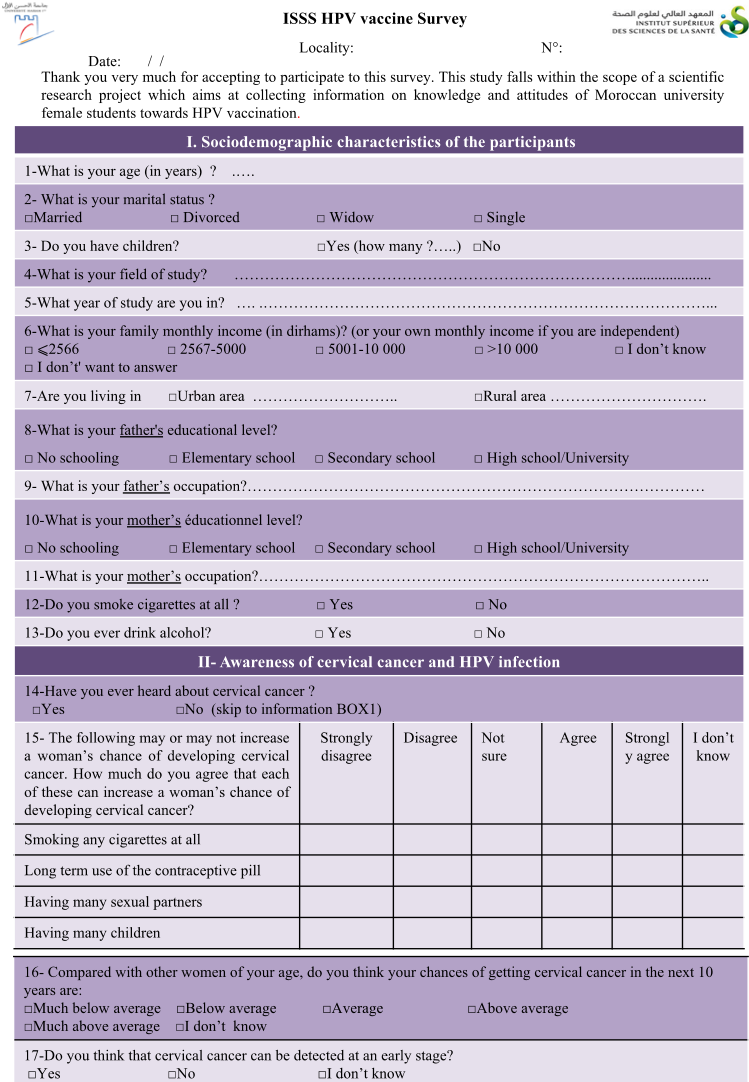


**S1 Fig.** Questionnaire used for data collection

**
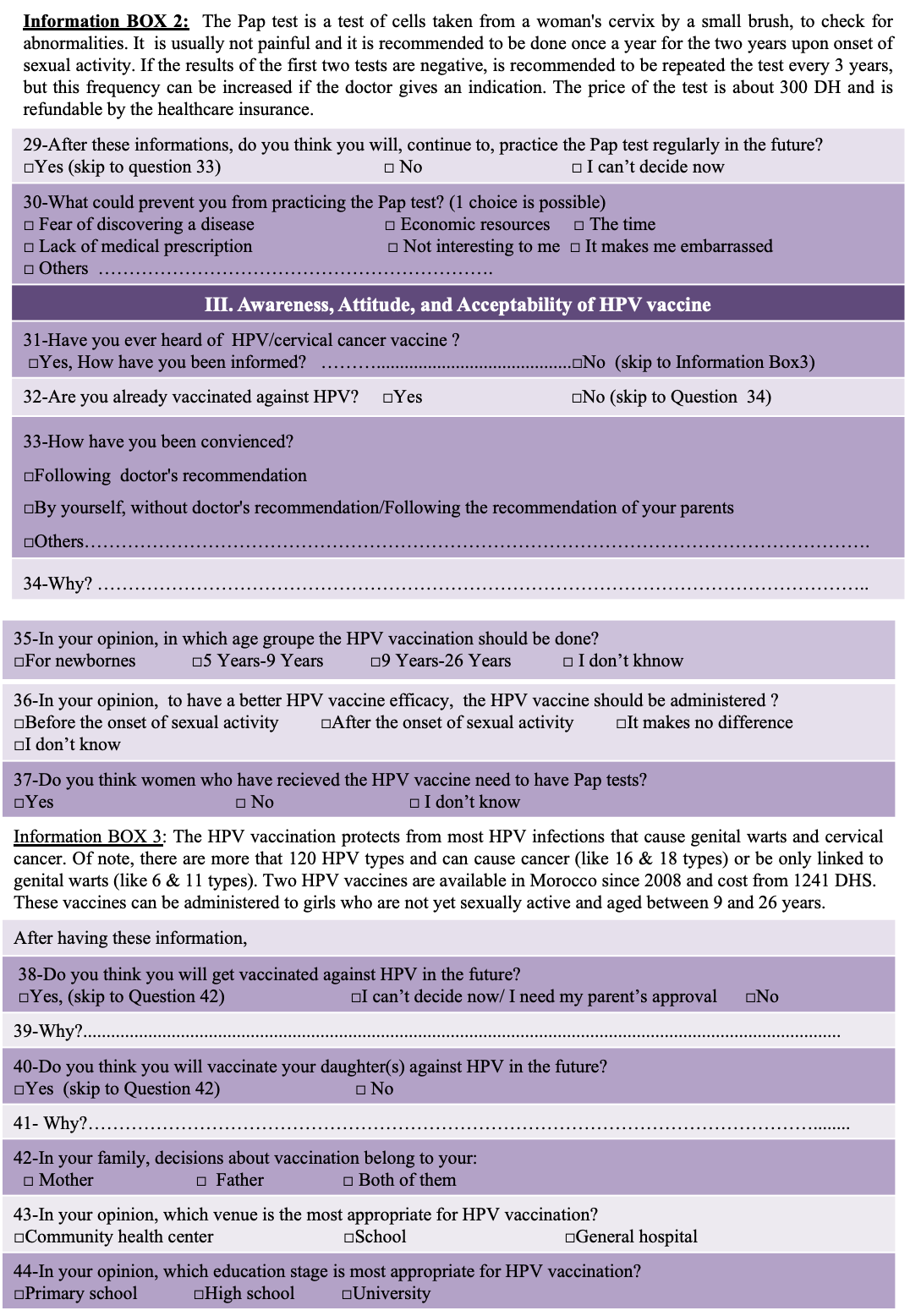
**

**S1 Fig. 2 (continued)**
